# DBSecSys 2.0: a database of *Burkholderia mallei* and *Burkholderia pseudomallei* secretion systems

**DOI:** 10.1186/s12859-016-1242-z

**Published:** 2016-09-20

**Authors:** Vesna Memišević, Kamal Kumar, Nela Zavaljevski, David DeShazer, Anders Wallqvist, Jaques Reifman

**Affiliations:** 1 Department of Defense Biotechnology High Performance Computing Software Applications Institute, Telemedicine and Advanced Technology Research Center, U.S. Army Medical Research and Materiel Command, Fort Detrick, MD 21702 USA; 2 Bacteriology Division, U.S. Army Medical Research Institute of Infectious Diseases, Fort Detrick, MD 21702 USA

**Keywords:** Bacterial secretion system, Virulence factors, Pathogenic mechanisms of action, Host-pathogen interactions, *Burkholderia mallei*, *Burkholderia pseudomallei*

## Abstract

**Background:**

*Burkholderia mallei* and *B. pseudomallei* are the causative agents of glanders and melioidosis, respectively, diseases with high morbidity and mortality rates. *B. mallei* and *B. pseudomallei* are closely related genetically; *B. mallei* evolved from an ancestral strain of *B. pseudomallei* by genome reduction and adaptation to an obligate intracellular lifestyle. Although these two bacteria cause different diseases, they share multiple virulence factors, including bacterial secretion systems, which represent key components of bacterial pathogenicity. Despite recent progress, the secretion system proteins for *B. mallei* and *B. pseudomallei,* their pathogenic mechanisms of action, and host factors are not well characterized.

**Results:**

We previously developed a manually curated database, DBSecSys, of bacterial secretion system proteins for *B. mallei*. Here, we report an expansion of the database with corresponding information about *B. pseudomallei*. DBSecSys 2.0 contains comprehensive literature-based and computationally derived information about *B. mallei* ATCC 23344 and literature-based and computationally derived information about *B. pseudomallei* K96243. The database contains updated information for 163 *B. mallei* proteins from the previous database and 61 additional *B. mallei* proteins, and new information for 281 *B. pseudomallei* proteins associated with 5 secretion systems, their 1,633 human- and murine-interacting targets, and 2,400 host-*B. mallei* interactions and 2,286 host-*B. pseudomallei* interactions. The database also includes information about 13 pathogenic mechanisms of action for *B. mallei* and *B. pseudomallei* secretion system proteins inferred from the available literature or computationally. Additionally, DBSecSys 2.0 provides details about 82 virulence attenuation experiments for 52 *B. mallei* secretion system proteins and 98 virulence attenuation experiments for 61 *B. pseudomallei* secretion system proteins. We updated the Web interface and data access layer to speed-up users’ search of detailed information for orthologous proteins related to secretion systems of the two pathogens.

**Conclusions:**

The updates of DBSecSys 2.0 provide unique capabilities to access comprehensive information about secretion systems of *B. mallei* and *B. pseudomallei*. They enable studies and comparisons of corresponding proteins of these two closely related pathogens and their host-interacting partners.

The database is available at http://dbsecsys.bhsai.org.

## Background

### Introduction

The highly infectious pathogen *Burkholderia mallei* is the causative agent of glanders, and its phylogenetically closest species, *B. pseudomallei*, is the causative agent of melioidosis. *B. mallei* evolved from an ancestral strain of *B. pseudomallei* by genome reduction and adaptation to an obligate intracellular lifestyle [1]. Given their considerable antibiotic resistance, ability to infect via aerosol exposure, and absence of vaccines, *B. mallei* and *B. pseudomallei* represent an emerging public health threat in their natural environment and as a potential bioterrorism threat. Although the two bacteria cause different diseases, they share multiple virulence factors, including bacterial secretion systems, which represent key components of bacterial pathogenicity [2, 3]. Recent research has provided new insights into *B. mallei* and *B. pseudomallei* pathogenicity [4, 5], but the identities of their secretion system proteins, their pathogenic mechanisms, and host factors are not completely characterized.

Several database systems provide general information about *Burkholderia* proteins [6–8], whereas more specific databases encompass information about single [9–12] or multiple [13, 14] bacterial secretion systems for many, but not all, *Burkholderia* species. In addition, existing databases of secretion systems for multiple pathogens provide general information about secretion system proteins of *B. mallei* and *B. pseudomallei* but lack specific information about protein functions and, especially, do not provide information about pathogenic mechanisms of action, experimental results, or host-interacting partners.

### Our contribution

To catalogue in-depth information about secretion system proteins of *B. mallei* and their host factors, we previously developed the Database of *Burkholderia mallei* Secretion Systems (DBSecSys) [15]. Since its deployment in July 2014, over 500 users from 38 countries from around the world have accessed DBSecSys. The users’ interest motivated us to update the database and extend it with information about secretion system proteins for *B. pseudomallei*. This updated version, DBSecSys 2.0, provides not only annotation for *B. mallei* and *B. pseudomallei* proteins associated with secretion systems but also manually curated information about these proteins, their involvement in virulence, their host targets (proteins), and their mechanisms of action inferred from literature and host-pathogen interaction data. These features provide an ability to study and compare secretion systems of the two pathogens not only through characterization of their corresponding pathogen proteins but also through characterization of their host-interacting partners. These data and features are currently not available elsewhere, and they make DBSecSys 2.0 a unique resource for *B. mallei* and *B. pseudomallei* secretion system research.

## Construction and content

### Database content

Table 1 shows the content of DBSecSys 2.0. Using the available literature on *Burkholderia* species and our experimental and computational work on *B. mallei* [16, 17], we compiled information for 204 *B. mallei* ATCC 23344 proteins and 281 *B. pseudomallei* K96243 proteins and their associated secretion systems, including 200 orthologous proteins. The majority of the proteins are associated with five secretion systems. In the updated database, we also included several recently identified secreted proteins that have been associated with virulence, but whose secretion mechanisms have not yet been determined (denoted as Undetermined Type in the database). We did not include information about contact-dependent interactions, which are involved in bacterial-bacterial interactions for *B. pseudomallei* [18], because the database focus is on secretion systems relevant for interactions with mammalian hosts.

**Table 1:** Summary of the content of DBSecSys 2.0

**Number of ** ***B. mallei***	
Proteins	204
Associated secretion systems	5
Virulence factors	52
Virulence attenuation experiments	82
Inferred mechanisms of action	12
**Number of ** ***B. pseudomallei***	
Proteins	281
Associated secretion systems	5
Virulence factors	61
Virulence attenuation experiments	98
Inferred mechanisms of action	13
**Number of host-pathogen protein-protein interactions**	
Human-*B. mallei* (experimental)	569
Murine-*B. mallei* (experimental)	788
Human-*B. mallei* (computational)	608
Murine-*B. mallei* (computational)	435
Human-*B. pseudomallei* (experimental)	4
Human-*B. pseudomallei* (computational)	1,117
Murine-*B. pseudomallei* (computational)	1,165

Published experimental data served as the main source of information for 82 virulence attenuation experiments for 52 *B. mallei* secretion system proteins and 98 virulence attenuation experiments for 61 *B. pseudomallei* secretion system proteins. For a subset of these experiments, we transferred virulence attenuation between orthologous proteins of the two pathogens. In these cases, we provided evidence that the inferred virulence attenuation is based on homology and, thus, needs experimental verification.

Since the publication of the first version of the database DBSecSys, there were no new published protein-protein interactions (PPIs) between *B. mallei* and human and murine hosts. Thus, we imported PPIs between *B. mallei* and 1,633 host proteins (795 human and 838 murine) from the previous version. Experimental host-pathogen PPI data for *B. pseudomallei* are scarce. We found only 4 published PPIs between *B. pseudomallei* and the human host [19]. To provide a more comprehensive picture of the host-pathogen PPIs for *B. pseudomallei*, we mapped host-pathogen PPIs for *B. mallei* proteins in the database to their orthologous *B. pseudomallei* proteins. PPIs between *B. mallei* protein and host proteins were identified previously in yeast two-hybrid (Y2H) experiments, i.e., they were based on direct protein interactions. Thus, the transfer of PPIs to orthologous *B. pseudomallei* proteins can be justified. The database now contains 2,400 host-pathogen PPIs between *B. mallei* and these 2 hosts and 2,286 host-pathogen PPIs between *B. pseudomallei* and these hosts. As previously described [15], DBSecSys 2.0 also contains a set of 491 PPIs [20] among 357 human proteins and 36 PPIs [21, 22] among 47 murine proteins that were identified as interacting partners of *B. mallei* proteins in Y2H experiments [16].

### Data sources

We identified secretion system proteins of *B. mallei* and *B. pseudomallei* using literature, experiments, orthology between *B. mallei* and *B. pseudomallei* proteins, and computational methods. For all experimental data and observations contained in DBSecSys 2.0, we provided links to their original sources and publications through PubMed.

Human-*B. mallei* and murine-*B. mallei* protein interaction data have been identified in Y2H experiments. As PPI detection experiments did not exhaustively screen for all possible host-*B. mallei* interactions, we used human-murine orthology information based on the HomoloGene [23] database to infer additional host-pathogen interactions, as previously described [15, 16]. Using these data, we transferred host-pathogen interaction data for *B. pseudomallei* to proteins that have orthologs to *B. mallei* proteins. We manually curated information about orthologous proteins between the two pathogens from the Kyoto Encyclopedia of Genes and Genomes (KEGG) pathways [24] and QuartetS_DB [25], including only bidirectional hits with a sequence identity larger than 95 %.

To account for the relationship among host proteins that interact with *B. mallei* and *B. pseudomallei* proteins, we used information from host PPI networks. For human PPIs, we used data from an experimentally detected PPI network compiled by Yu et al. [20], whereas for murine PPIs, we used data from an experimentally detected PPI network available in the BioGRID database (release 3.2.105) [21, 22].

We inferred pathogen mechanisms of action for proteins in the database using common terms from the available literature shown in Table 2. To better account for recent research about the two pathogens, the list of mechanisms of action was extended with 3 additional terms, cytotoxicity, intracellular survival, and regulation. For *B. mallei* proteins, we used mechanisms of action based on the literature, Y2H experiments, and additional computations, as previously described [15]. For *B. pseudomallei* proteins, we used the literature as the primary source of information about mechanisms of action. To provide additional information about possible mechanisms of action, we assigned all mechanisms of action bi-directionally between orthologous proteins of *B. mallei* and *B. pseudomallei*.

**Table 2:** Description of pathogenic mechanisms of action included in DBSecSys 2.0

Name	Pathogens use this mechanism to:
Actin cytoskeleton rearrangement	Subvert the host cell cytoskeleton to promote attachment to the host cell surface, internalization in the host cell, and prevent uptake by phagocytic cells.
Actin-based motility	Bind to host actin, triggering actin polymerization on the pathogens’ surface and producing a mechanical force that propels them through the host cell and facilitates cell-to-cell spread.
Adhesion	Attach to the host cell surface, promoting bacterial internalization in the host cell.
Apoptosis	Exert control on the processes that regulate apoptosis in the host.
Cytotoxicity^a^	Secrete toxins into the host cell.
Interference with signaling	Interfere with the host signaling cascade, promoting their internalization in the host cell and intracellular survival.
Interference with the immune response	Downregulate host inflammatory responses, promoting their internalization in the host cell and intracellular survival.
Intracellular survival^a^	Evade the host immune response and multiply in the host cell.
Invasion	Promote their ability to invade the host cell.
Multinucleated giant cell formation	Induce host cell fusion and multinucleated giant cell formation.
Phagosomal escape and evasion of autophagy	Ensure bacterial escape from endocytic vesicles as well as to evade autophagosomes, ensuring the pathogens’ intracellular survival and cell-to-cell spread.
Regulation^a^	Control secretion system activation and related mechanisms of pathogenicity.
Ubiquitination–deubiquitination	Interfere with host ubiquitination processes to attenuate the host immune response, to prevent their degradation, and to ensure their destruction when no longer required for establishing the infection.

^a^Mechanisms of action added in the updated database

We used the National Center for Biotechnology Information (NCBI) [23] and Uniprot [26] to annotate pathogen and host proteins, e.g., protein names and sequence information. For functional annotation of host proteins, we used Gene Ontology (GO) terms [27] and KEGG pathways [24].

### Software architecture

In DBSecSys 2.0 database and Web interface, we used a three-tier architecture, which we developed for the previous version [15]: *1*) a backend Oracle database server that stores the data contained in the DBSecSys 2.0 database, *2*) the controller, and *3*) the presentation tier. We used Java Platform, Enterprise Edition 7, JavaServer Faces 2, and ICEfaces 3 technologies. The presentation tier also includes JBrowse [28], D3.js [29], NVD3.js [30], and Cytoscape.js [31] JavaScript libraries that provide detailed interactive visualizations of genes on the reference sequence and of PPIs. For DBSecSys 2.0, we updated the data access layer to provide a more consistent and faster user experience. Some of the searches are 2 to 3 time faster than the previous version. We have also added a loading indicator to provide visual feedback for any long running searches. The DBSecSys 2.0 Web application is hosted on an Apache Tomcat Web server at http://dbsecsys.bhsai.org.

## Utility and Discussion

Users can browse and query cross-linked data in DBSecSys 2.0 through a Web-based graphical user interface, which supports five functionalities: query for *1*) pathogen protein or host protein annotation, *2*) pathogen proteins associated with a secretion system, *3*) pathogen proteins associated with the pathogen’s mechanism of action, *4*) host-pathogen interactions, and *5*) experimentally screened virulence factors. We provided details of these functionalities for a single organism in the previous version of the database, which have been updated in the current User’s Guide. Users can download query results in a tab-delimited format and host-pathogen PPIs in the Proteomics Standards Initiative Molecular Interactions (PSI-MI) Tab 2.5 format [32]. DBSecSys 2.0 also provides links to external resources for detailed information about individual proteins (NCBI, Uniprot, GO, and KEGG), as well as links to relevant publications in PubMed.

Importantly, the new database content and design enable comparative analysis between organisms. We describe two such applications below.

### *Application 1*: search for orthologous proteins in *B. mallei* and *B. pseudomallei*

Users can study individual (pathogen or host) proteins on the “Proteins” page of the Web interface. Users can search for association of a pathogen protein with a secretion system using standard protein/gene identifiers, such as Locus Tag, Name, or Uniprot ID. When the ortholog of the query protein is present in the database, DBSecSys 2.0 provides a link to it. For example, a search by Name for the virulence factor TssM [19, 33] returns links to orthologous proteins in the two organisms. Users can click one of the links, e.g., the link to *B. mallei*, to display the protein page with detailed annotation information about this protein, including its Locus Tag BMAA0729, evidence about its association with the type 2 secretion system (T2SS), virulence attenuation experiments, inferred mechanisms of action, and host-pathogen interactions. Selecting the box labeled “Pathogen Ortholog Comparison” and clicking the link for BMAA0729, the database returns information about TssM’s orthologous protein in *B. pseudomallei*, with Locus Tag BPSS1512, on the lower half of the screen. This enables direct comparison of annotations for the two orthologs. The information about orthologs is very similar because of the database curation strategy to transfer virulence information, mechanisms of action, and host-pathogen interactions between two orthologous proteins. Comparison between secretions systems of these orthologous proteins provides additional insights.

### *Application 2*: comparison of related secretion systems in *B. mallei* and *B. pseudomallei*

**Fig. 1:**
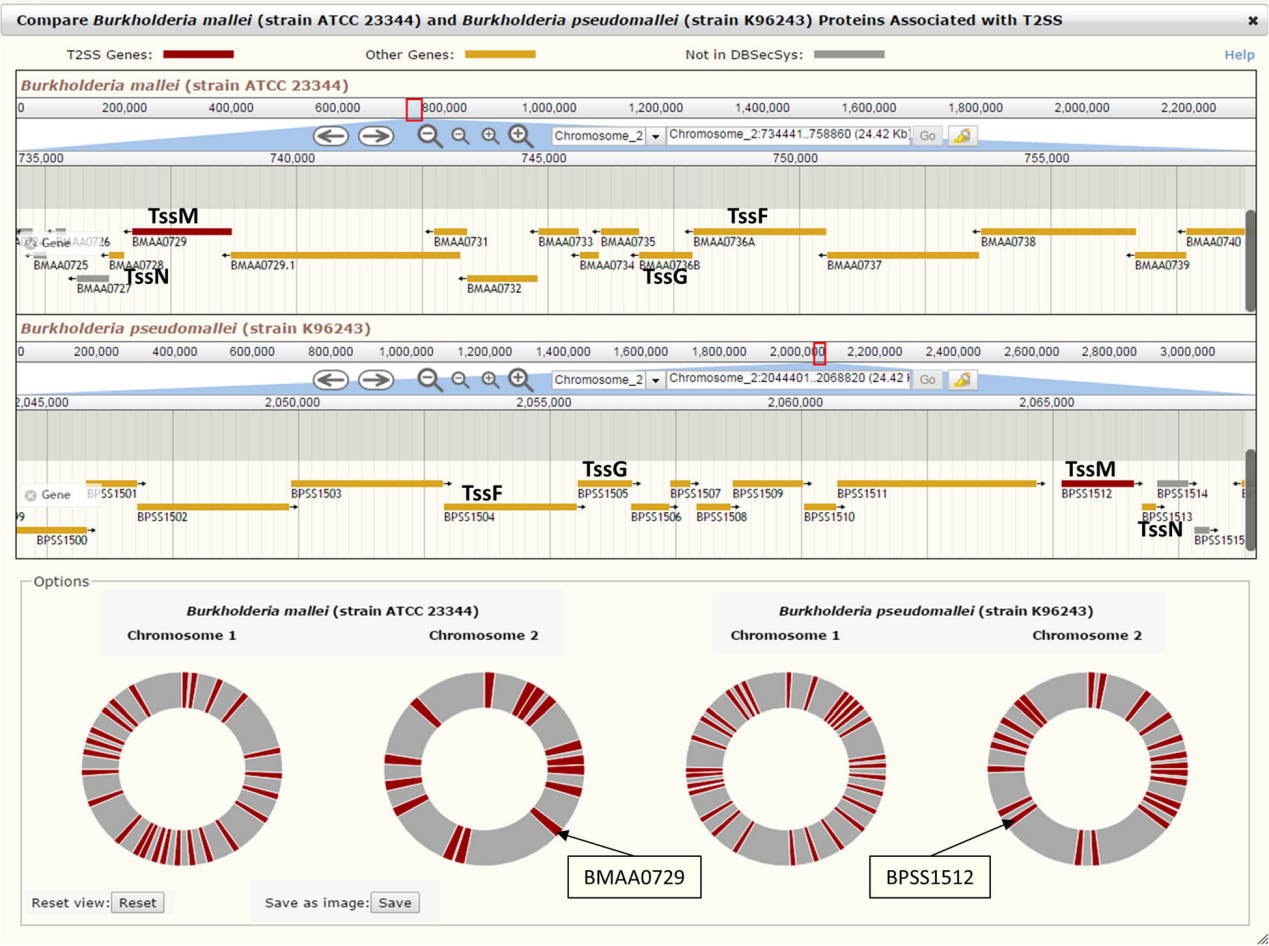
Comparison of related secretion systems in *Burkholderia mallei* and *B. pseudomallei.* The genome browser shows the location of the orthologous proteins TssM, TssN, TssF, and TssG and their association with related secretion systems on the bacterial reference sequences for *B. mallei* strain ATCC 23344 and *B. pseudomallei* strain K96243. Type 2 secretion system (T2SS) and type 6 secretion system (T6SS) proteins are depicted in red and yellow, respectively

Users can study pathogen proteins associated with a specific secretion system on the “Secretion Systems” page of the Web interface. Continuing the previous example, users can compare secretion systems for two TssM proteins by clicking the link to T2SS and selecting the link “Compare *Burkholderia mallei* (strain ATCC 23344) and *Burkholderia pseudomallei* (strain K96243)” from the drop-down list “Select Pathogen.” The lists of proteins in the corresponding secretion systems of the two pathogens are displayed on the same page and enable direct comparison between individual proteins. In addition, users can open the link to the genome browser, which depicts the positions of these proteins on the genomes of the two organisms, including linear genome maps and schematic circular chromosomes. Positioning the mouse over the marked red bands on the circular chromosomes shows individual proteins or secretion system clusters. Clicking at the positions on chromosome 2 of each organism corresponding to TssM, BMAA0729 in *B. mallei* and BPSS512 in *B. pseudomallei*, shows their positions on the corresponding linear genome maps. In addition, by adjusting the coverage of the genomic regions, users can visualize the position of this T2SS protein relative to the adjacent type 6 secretion system (T6SS) proteins in *B. mallei* and *B. pseudomallei*, as shown in Fig. 1. Users can inspect the syntenic regions for the two pathogens to visualize orthologous pairs. For examples, the nearest neighbor of TssM is TssN, which is shown at Locus Tags BMAA0728 in *B. mallei* and BPSS1513 in *B. pseudomallei*. Previously, we found that both transposon insertion and deletion of this gene in *B. mallei* fully attenuate virulence [16, 34]. Because of its importance for virulence and its regulation by the same mechanisms as cluster 1 T6SS [35–37], an important virulence factor, we putatively assigned TssN to T6SS.

Another interesting protein is TssF, with Locus Tag BPSS1504 in *B. pseudomallei*, which was recently identified as an important virulence factor for *B. pseudomallei* [38]. Neither NCBI nor KEGG provided a *B. mallei* ortholog for this protein, because the corresponding genomic region was originally annotated as a pseudogene [39]. However, experimental evidence indicated that this region in *B. mallei* codes for two proteins, designated as BMAA0736A and BMAA0736B [35]. Based on the annotation from the Pathosystems Resource Integration Center (PATRIC) [6], these two proteins are orthologs to TssF and TssG, respectively, in *B. pseudomallei*, and their protein sequences in *B. mallei* have the same length and 99 % sequence identity to the corresponding *B. pseudomallei* orthologs. We included these sequences in DBSecSys 2.0, providing for a more comprehensive comparison of cluster 1 T6SS for the two pathogens.

### Database updates

The database content will be expanded with information for additional pathogens and will be updated periodically. Before each update, the current state of the database will be frozen and archived, and can be requested by contacting dbsecsys@bhsai.org.

## Conclusions

We previously developed DBSecSys, a database of *B. mallei* secretion system proteins. The database organization provided capability to store and retrieve comprehensive information about pathogen proteins associated with secretion systems, such as their annotation, involvement in virulence, host protein targets, and mechanisms of action inferred from the literature and host-pathogen interaction data. In DBSecSys 2.0, we improved data querying and retrieval, enabling comparison of secretion systems for multiple pathogens. In addition, we expanded the database with new information about *B. pseudomallei* and updated the information about *B. mallei*. These updates provide unique capabilities to access comprehensive information about the secretion systems of *B. mallei* and *B. pseudomallei*. Importantly, they enable studies and comparisons of corresponding proteins of these two closely related pathogens and their host-interacting partners.

## Abbreviations

DBSecSys: Database of *Burkholderia mallei* and *Burkholderia pseudomallei* secretion systems
GO: Gene ontology
KEGG: Kyoto encyclopedia of genes and genomes
NCBI: National Center for Biotechnology Information
PATRIC: Pathosystems resource integration center
PPI: Protein-protein interaction
Y2H: Yeast two-hybrid
T2SS: Type 2 secretion system
T6SS: Type 6 secretion system
